# Response to joint selection on germination and flowering phenology depends on the direction of selection

**DOI:** 10.1002/ece3.4334

**Published:** 2018-07-12

**Authors:** Laura F. Galloway, Ray H. B. Watson, Holly R. Prendeville

**Affiliations:** ^1^ Department of Biology University of Virginia Charlottesville Virginia; ^2^ Present address: USDA FS Pacific Northwest Research Station Corvallis Oregon

**Keywords:** artificial selection, bivariate selection, *Campanula americana*, *Campanulastrum americanum*, correlated response, flowering time, germination time, life history evolution, maternal effects, realized heritability, reproductive phenology

## Abstract

Flowering and germination time are components of phenology, a complex phenotype that incorporates a number of traits. In natural populations, selection is likely to occur on multiple components of phenology at once. However, we have little knowledge of how joint selection on several phenological traits influences evolutionary response. We conducted one generation of artificial selection for all combinations of early and late germination and flowering on replicated lines within two independent base populations in the herb *Campanula americana*. We then measured response to selection and realized heritability for each trait. Response to selection and heritability were greater for flowering time than germination time, indicating greater evolutionary potential of this trait. Selection for earlier phenology, both flowering and germination, did not depend on the direction of selection on the other trait, whereas response to selection to delay germination and flowering was greater when selection on the other trait was in the opposite direction (e.g., early germination and late flowering), indicating a negative genetic correlation between the traits. Therefore, the extent to which correlations shaped response to selection depended on the direction of selection. Furthermore, the genetic correlation between timing of germination and flowering varies across the trait distributions. The negative correlation between germination and flowering time found when selecting for delayed phenology follows theoretical predictions of constraint for traits that jointly determine life history schedule. In contrast, the lack of constraint found when selecting for an accelerated phenology suggests a reduction of the covariance due to strong selection favoring earlier flowering and a shorter life cycle. This genetic architecture, in turn, will facilitate further evolution of the early phenology often favored in warm climates.

## 1 INTRODUCTION

Selection on a single trait may result in the evolution of multiple traits, as trait correlations are pervasive due to shared genetic, developmental, functional, or environmental associations (Conner & Hartl, 2004; Peiman & Robinson, 2017). The strength and orientation of genetic correlations, in conjunction with the pattern of selection, can enhance or constrain evolutionary response (Agrawal & Stinchcombe, 2009; Conner et al., 2011; Lande, 1979; Simonsen & Stinchcombe, 2010; Teplitsky et al., 2014; Walling et al., 2014). For instance, if selection is in the same direction for two traits, a negative correlation between them will slow the adaptive response, while a positive correlation would accelerate it (Conner, 2012). However, genetic correlations between traits may vary among populations, environments, or even different portions of the trait distributions (Wood & Brodie, 2015), suggesting that their contribution to evolution is more variable than often appreciated.

A majority of empirical studies have demonstrated that cross‐generation genetic correlations tend to be negative and act in opposition to within‐generation genetic correlations (Galloway, Etterson, & McGlothlin, 2009; Räsänen & Kruuk, 2007; Wilson & Réale, 2006). Opposing maternal–offspring genetic correlations can act as a constraint to rapid evolutionary change as they reduce the available additive genetic variation and as such may be favored during episodes of stabilizing selection (Wolf & Brodie, 1998). Life history traits are expected to be under stabilizing selection due to their close relationship with fitness. Consequently, rapid responses to fluctuating directional selection may be maladaptive, and the constraint imposed by opposing maternal–offspring genetic correlations may help slow phenotypic evolution in unstable environments (Hoyle & Ezard, 2012). While the theoretical impacts of genetic correlations within and between generations are well described, the extent to which genetic correlations enhance or constrain the evolutionary response of traits that are functionally linked across generations is little known.

Frequently traits may be correlated through several mechanisms. In particular, life history traits that comprise complex phenotypes such as phenology (Armbruster, Pélabon, Bolstad, & Hansen, 2014; Murren, 2012; Peiman & Robinson, 2017), may have both genetic and functional linkages. For example, in *Arabidopsis thaliana*, the FLOWERING LOCUS C (FLC) gene regulates flowering time and mediates germination time by affecting seed dormancy through a pleiotropic genetic correlation (Chiang et al., 2009). Simultaneously, in *A. thaliana* and many flowering plants, the sequential nature of expression of phenological traits results in functional linkage, as the timing of flowering affects traits later in development such as fruit maturation and seed dispersal (Donohue, 2009; Galloway & Burgess, 2009; Lacey & Pace, 1983; Lacey, Roach, Herr, Kincaid, & Perrott, 2003). These correlations may extend into the next generation such that timing of flowering influences offspring germination environment by determining the timing of seed dispersal. However, we have little knowledge of how the evolutionary response to selection of individual components of complex phenotypes is affected by this array of correlations.

Here, we evaluate whether correlations between phenological traits that determine life history schedule make some evolutionary outcomes more likely than others. In the herb *Campanula americana*, germination season determines life history schedule with fall germinants flowering the following summer as annuals, while spring germinants flower in their second year as biennials (Baskin & Baskin, 1984). Season of germination is influenced by the seed's genetics as well as by maternal flowering time. Early‐flowering plants disperse seeds early, increasing the potential for fall germination of offspring, while seeds of late‐flowering plants are frequently dispersed in late fall when it is cool and are therefore more likely to germinate the following spring (Galloway, 2002; Galloway & Burgess, 2009, 2012). We predict that there is a genetic correlation between timing of germination and flowering, so they provide a coordinated influence on life history schedule (Peiman & Robinson, 2017). Furthermore, we predict that the correlation is negative as the traits represent maternal and offspring influences on life history schedule. However, work to date provides little evidence for a genetic correlation between these traits (Galloway et al., 2009; Prendeville, Barnard‐Kubow, Dai, Barringer, & Galloway, 2013), raising questions as to whether the predicted relationships will be found. As both germination and flowering time affect life history schedule, this system provides a compelling example of how selection on specific traits in a complex phenotype such as phenology may have ramifications for other components of the phenotype.

We evaluate possible correlations between phenological traits that determine life history schedule by conducting artificial selection on the two components of phenology simultaneously. Artificial selection is a powerful tool for elucidating the contribution of trait correlations to evolution (Berner, 2012; Conner, 2003). However, joint (bivariate) selection is rarely used. Joint artificial selection mimics selection in nature that typically occurs on multiple traits at once. It can uncover the contribution of correlations to the evolution of multiple traits that are under different patterns of selection, a scenario that is likely for traits that comprise a complex phenotype. We selected germination and flowering time in the same direction (both early and both late) or in opposite directions (early germination and late flowering and vice versa). We then measured the response to selection and realized heritability for each trait and determined whether evolution differed depending on whether selection on the other trait was in the same or opposing direction. We use these results to inform our understanding of how trait interactions affect evolution of phenology and life history schedule.

## 2 MATERIALS & METHODS

### 2.1 Study system

*Campanula americana* L. (=*Campanulastrum americanum* Small; Campanulaceae) is an outcrossing, autotetraploid herb with a geographic range spanning the Eastern United States (Barnard‐Kubow, Debban, & Galloway, 2015; Galloway, Etterson, & Hamrick, 2003). Seeds germinate in the fall or spring, and vernalization is required to induce flowering; thus, fall germination and spring germination, respectively, result in annual and biennial life histories. Flowering begins in mid‐summer and fruits ripen and dehisce throughout the fall. Seeds disperse when fruits mature (Galloway & Burgess, 2009) and are nondormant at maturity (Baskin & Baskin, 1984), so the reproductive phenology of maternal plants directly influences offspring germination season (Galloway, 2002; Galloway & Burgess, 2009, 2012). Germination and flowering time in *C. americana* have significant additive genetic variation (Galloway et al., 2009). Flowering time displays substantial evolution in response to artificial selection (Burgess, Etterson, & Galloway, 2007) and is more variable among populations than timing of germination (Prendeville et al., 2013).

### 2.2 Artificial selection

**Figure 1:**
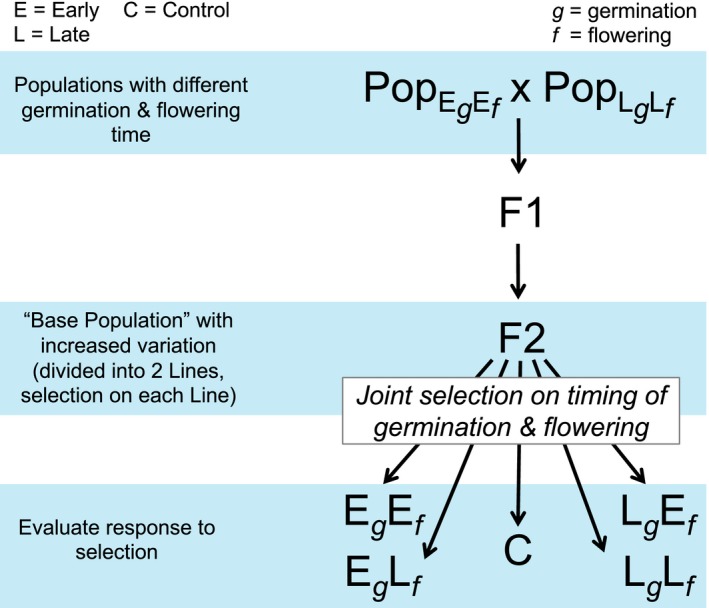
Schematic of design of selection experiment. A population with relatively early germination and flowering time was crossed to one with late germination and flowering. F1s were grown and crossed to form a variable F2 generation to serve as a base population for selection. Base populations were divided into two replicate lines. Each line was selected for the four combinations of early and late germination and flowering time as well as a control, where individuals were selected at random. Germination and flowering time were scored on offspring of F2 to determine response to selection. The process was repeated for two pairs of populations, creating independent replicates to assess the response to selection

To determine the consequences of joint selection on germination and flowering time, we initially created base populations with increased variation for the two traits. Seeds collected from 33 populations that spanned the latitudinal extent of the geographic range were grown in the greenhouse for two generations, and germination and flowering time were recorded (Prendeville et al., 2013). Two pairs of populations, each with one relatively early‐germinating, early‐flowering population and one late‐germinating, late‐flowering population (Table S1), were selected to form base populations with increased genetic variance that could be exploited in artificial selection (Figure 1). Base Population I comprised populations that diverged in germination and flowering time across the latitudinal range (1,195 km apart). It included an “early” population from the north (MI44) whose seeds germinated in 13.0 days and plants flowered 75 days postvernalization and a “late” population from the south (MS55) with germination in 14.3 days and flowering 102 days after vernalization. Base Population II was created from geographically fairly close populations (308 km apart). The early population (TN34) seeds germinated in 11.4 days and flowered 59 days after vernalization, while late population (MS70) seeds germinated in 18.5 days and plants flowered 84 days after vernalization.

The populations within each pair were crossed to produce an F1 with an intermediate timing of germination and flowering (Figure 1). In the field, seeds were collected by maternal plant, with the number of maternal seed families varying somewhat among populations (Base Population I: MI44 = 29, MS55 = 19; Base Population II: TN34 = 28, MS70 = 8). For all populations, 10 seeds were planted for each family in plug trays filled with a soil‐free mix (3 Promix BX: 1 turface) and germinated in a growth chamber (25°C days/14°C night, 12 hr days). Seedlings were thinned to 30 per population, evenly distributed across families, and vernalized at 5°C for 7 weeks (12 hr days) to induce flowering. Plants were then transplanted into 4 × 14 cm tubular pots and moved to a greenhouse where they were randomly located, watered regularly, fertilized every 2 weeks until bolting and then weekly thereafter, and grown under supplemental lighting (16 hr day). When flowering, crosses were conducted between populations within each pair. For each population, 25 individuals served as pollen recipients for two crosses, each from a different sire from the other population, resulting in 50 crosses/population and 100 F1 families for each base population. Flowers were emasculated prior to pollination. Seed was collected when fruits matured.

F1 families were crossed to create variable F2 base populations for artificial selection (Figure 1). Eight seeds from each F1 family were planted, thinned to one seedling, and grown as described above. Each F1 plant was crossed to plants of the same type, serving once as a pollen donor and once as a recipient, resulting in 100 F2 families in each base population.

Germination and flowering time were scored for the F2 generation to determine selection groups. Ten seeds from each F2 family (100 families/base population x two base populations = 2,000 seeds) were planted singly in random order in germination trays during the fall germination season (3–4 Oct.). Seed trays were placed outdoors near the UVA greenhouse, and germination was scored daily for 36 days until it had nearly stopped. In nature, cold winter temperatures interrupt germination, so late‐germinating seeds emerge in the spring as biennials. However, the mild conditions outside the greenhouse resulted in most seeds germinating soon after planting (86.4%). Therefore, we measure timing of germination in days rather than seasons. Unfortunately, plantings into the ground under field conditions were not successful. Seedlings were vernalized, and then 1,200 plants, distributed evenly across families (600 per base population), were transplanted and grown to flowering in the greenhouse as described above. Flowering was scored daily until all plants had flowered.

Artificial selection for all combinations of early and late germination and early and late flowering was conducted on F2 plants (Figure 1). Each base population was randomly split into two replicate lines of around 300 plants. For each line, the 20 individuals with the earliest germination and earliest flowering times were selected (6.7%). While the joint selection was strong, selection on the traits individually would have been stronger as selected plants were not necessarily the earliest to germinate or flower. Crosses among these selected plants formed the early germination/early flowering selection treatment, that is, “E_*g*_E_*f*_.” Plants were crossed to unrelated members of the selected group, serving as a pollen donor to two plants and a pollen recipient for two other plants, creating 40 E_*g*_E_*f*_ families. This procedure was repeated for the 20 latest germinating and latest flowering plants, “L_*g*_L_*f*_” selection treatment, as well as early germinating and late flowering “E_*g*_L_*f*_,” and the converse “L_*g*_E_*f*_.” Control groups (“C”) were created by crossing 20 randomly chosen individuals for each line and base population (Figure 1). In total, there were two replicate lines of the four germination/flowering time selection treatments and control in each of the two base populations for a total of 20 lines (five selection treatments x two lines/base population x two base populations). Seed was collected when mature.

The following growing season, response to selection on germination and flowering time was determined for offspring of selected and control treatments. A total of 2,400 seeds were planted and placed outdoors using the same procedure and at the same time of year as the F2 generation (7 Oct.). These included three seeds for each of the 40 families in each line and base population (three seeds/family × 40 families x two lines x two base populations = 480 seeds for each of the five selection treatments: E_*g*_E_*f*_, E_*g*_L_*f*_, L_*g*_E_*f*_, L_*g*_L_*f*_, C). Germination was scored daily for 53 days at which time it had nearly stopped, resulting in 86.6% germination. Timing of germination was measured as number of days until germination. Eighty randomly selected plants were transplanted for each selection treatment, line, and base population combination. Plants were vernalized and then grown in the greenhouse as described above. Flowering was scored daily, and timing of flowering was measured as number of days from vernalization until first flower. Plants were kept in the same order throughout the experiment, and germination tray was included in analyses as a blocking factor (“block”).

### 2.3 Statistical analysis

Response to selection on germination and flowering time was evaluated using a generalized linear mixed model with selection treatment, base population, and their interaction as fixed effects and line (nested within base population) and block as random effects. A significant interaction between selection treatment and base population would indicate that the response to selection differs between the base populations. A log‐normal distribution with an identity link was assigned for both germination and flowering time to meet model assumptions (PROC GLIMMIX, SAS v. 9.4). To evaluate response to selection for germination and flowering time, a priori contrasts were performed in each base population between each selection treatment and the control. To determine whether response to selection on one trait depended on the pattern of selection at the other trait, contrasts were performed between the treatment that experienced selection in the same direction for both traits, for example, E_*g*_E_*f*_, and the treatment that had been selected in opposite directions, for example, E_*g*_L_*f*_ for germination or L_*g*_E_*f*_ for flowering. Contrasts were conducted on selection for early and late germination and flowering in both base populations.

Realized heritability of germination and flowering time were calculated for each selection treatment and line in the two base populations. Realized heritability (*h* ^*2*^) is the response to selection (*R*) divided by the selection differential (*S*). *S* was calculated for each line and base population as the difference between the mean of each F2 selection treatment and the F2 population mean. *R* was calculated for each selection treatment as the difference between the means of the postselection generation and the F2‐selected individuals, again for each line and base population. To remove potential cross‐generation differences in environmental conditions, the control treatment was averaged across lines within a base population for each generation and that value was subtracted from the mean of each selection treatment for that generation when calculating *R*. To account for variance due to drift, standard errors (*SE*) for each estimate of *h* ^*2*^ were calculated as follows: $\text{Var}\left(h^{2}\right)\approx V_{p}/S^{2}\left[(h^{2}/N\right)+\left(2/M\right)]$ where *V*_*p*_ is the within‐generation phenotypic variance, *M* the number of plants measured with that generation, and *N* the number of plants selected as parents of the next generation (Roff, 1997). Zero was substituted in the formula for *h* ^*2*^ when it was negative.

## 3 RESULTS

**Figure 2:**
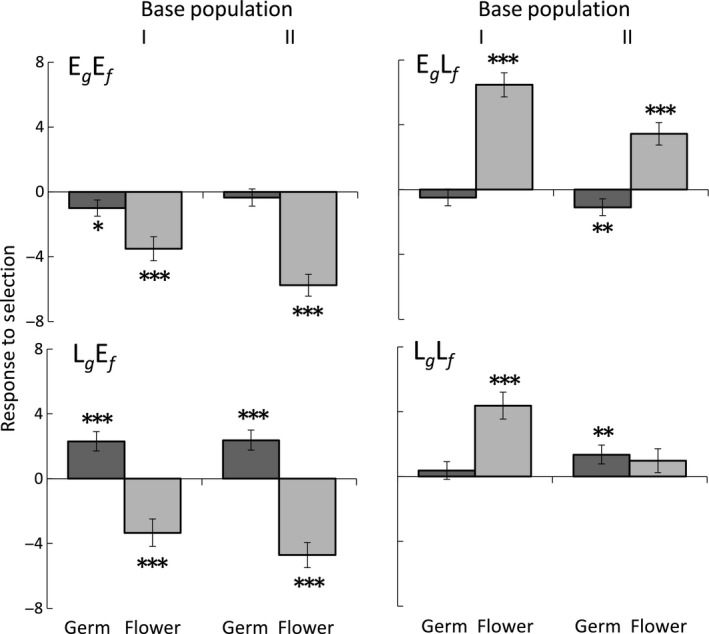
Deviation of the mean number of days to germination and flower of each selection treatment from the mean of the control (±*SE*) in *Campanula americana* following one generation of artificial selection for early germination and early flowering (E_*g*_E_*f*_), early germination and late flowering (E_*g*_L_*f*_), late germination and early flowering (L_*g*_E_*f*_), and late germination and late flowering (L_*g*_L_*f*_). Means are the average of two replicate lines for each base population. Asterisks indicate results of contrasts comparing each selection treatment with the control. *p ≤* *0.05, **p ≤* *0.01, ***p ≤* *0.001

There was a greater response to selection for timing of flowering than timing of germination. Following selection, there was an average 2‐day difference between the early and late selection lines in germination time, representing a change of up to 0.63 standard deviations, with five of eight treatments differing significantly from the control (Table 1, Figure 2). The largest difference was for the late germination/early flowering (L_*g*_E_*f*_) treatment in each base population, with a delay in germination greater than 0.85 *SD* of the control means. There was a trend (*p* < 0.08, Table 1) of a difference in response to selection between the base populations for germination time that was largely driven by the greater response of the late germination/late flowering (L_*g*_L_*f*_) treatment in Base Population II compared to Base Population I (Figure 2). Following selection, flowering time differed by an average of 8 days between the early and late selection lines, a change of as much as 0.89 standard deviations, with seven of the eight treatments significantly different from the control (Table 1, Figure 2). Only flowering time of the late germination/late flowering (L_*g*_L_*f*_) treatment in Base Population II did not show a significant response to selection. Response differed between the base populations (Table 1), with a greater response to selection for late flowering in Base Population I and for early flowering in Base Population II (Figure 2).

**Table 1:** Analysis of variance comparing days to germination and days to flowering of *C. americana* selected for all combinations of early and late germination time and flowering time, as well as a control treatment

Source	Num *df*	Germination *F*/*Z*	Flower *F*/*Z*
Selection treatment	4	35.17***	141.32***
Base population	1^a^	1.75	139.12**
Selection*Base pop	4	2.10+	3.14*
Line(Base pop)		0.91	0.65
Block		1.95*	2.71**
Error *df*		1,581	1,581
Contrast: Does response depend on pattern of selection on the other trait?			
Early selection: Base pop I	1	1.75	0.07
Early selection: Base pop II	1	3.29+	2.84+
Late selection: Base pop I	1	18.49***	6.96**
Late selection: Base pop II	1	4.79*	12.54***

*Notes*

Artificial selection was conducted on two base populations, each an F2 from a between‐population cross, and two lines within each base population. Contrasts between levels of the selection treatment permit evaluation of whether response to selection was influenced by the direction of selection on the other trait. F‐values are reported for fixed effects and Z‐values for the random effects of line and block, ^+^ *P* < 0.10, *p < 0.05, **p < 0.01, ***p < 0.001.

^a^Base population error *df* = 2.

The influence of trait correlations on evolution depended on the direction of selection. For both germination and flowering, there were stronger responses for late selection when it was paired with early selection on the other trait (Table 1, Figure 2: germination L_*g*_E_*f*_ vs L_*g*_L_*f*_, flowering E_*g*_L_*f*_ vs L_*g*_L_*f*_). In contrast, for both traits, response to selection for earlier phenology did not depend on the pattern of selection on the other trait (Table 1).

**Figure 3:**
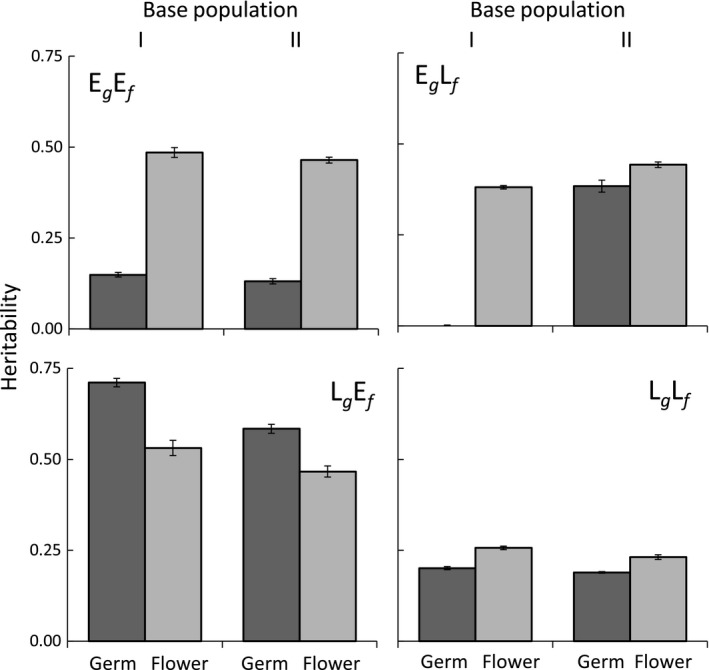
Realized heritability (±*SE*) of days to germination and days to flowering in *Campanula americana* following artificial selection for early germination and early flowering (E_*g*_E_*f*_), early germination and late flowering (E_*g*_L_*f*_), late germination and early flowering (L_*g*_E_*f*_), and late germination and late flowering (L_*g*_L_*f*_) averaged across two replicate lines for each of the base population

With the exception of early flowering, realized heritabilities were generally larger when selection was in opposite directions on the two traits. Realized heritability for days to germination was generally low ($\overset{¯}{x}=0.17$) for treatments where selection on traits was in the same direction (e.g, E_*g*_E_*f*_ and L_*g*_L_*f*_; Figure 3; Table S2) but generally high ($\overset{¯}{x}=0.56$) where selection was in opposite directions (E_*g*_L_*f*_ and L_*g*_E_*f*_). The exception to this was E_*g*_L_*f*_ Base Population I with an average heritability of zero (Table S2, Figure 3). The pattern of higher realized heritability when selection was in the opposite direction on the two traits was also found for late flowering ($\overset{¯}{x}=0.46$; Figure 3). However, realized heritability for early flowering was almost the same, regardless of selection on germination timing ($\overset{¯}{x}=0.47$; Figure 3). Patterns were largely consistent across base populations.

## 4 DISCUSSION

Selection was conducted on all four combinations of early and late germination and flowering time in *Campanula americana*. Flowering time had a large response to selection, with an average 8‐day difference between the early‐ and late‐flowering selection treatments, whereas germination time had a more modest response, with only an average 2‐day difference. However, there was a greater response to selection for later phenology, later germination and later flowering, when selection was in opposite directions on the two traits (e.g., early germination and late flowering) than when selection was in the same direction (e.g., late germination and late flowering), indicating that a correlation between the traits affected their evolution. Response to selection for earlier phenology, germination or flowering was not affected by the direction of selection on the other trait, indicating that traits were independent in their evolution. This pattern of evolution, where direction of selection determined whether trait correlations affected the response, was found for both traits and in two independent base populations and supports a conserved genetic architecture among populations. Further, it reveals variation in the extent to which components of phenology expressed across the life cycle are correlated and thus function as a complex phenotype.

Greater response to selection and realized heritability for flowering time than timing of germination in *C. americana* are in keeping with other taxa. Over both base populations, realized heritability for flowering time (median *h* ^2 ^= 0.42) was greater than that for germination time (median *h* ^2 ^= 0.27). Early life cycle traits typically have smaller heritability than those expressed at later stages (reviewed in Geber & Griffen, 2003 [*h* ^2 ^= 0.38 flowering time, *h* ^2 ^= 0.11 germination time]; Simons & Johnston, 2006). Substantial maternal contributions early in the life cycle likely underlie this pattern (Galloway et al., 2009; Wilson & Réale, 2006). The observed response to selection for flowering time here is commensurate with previous work in a different *C. americana* population where three generations of artificial selection resulted in a divergence of 25 days between early‐ and late‐flowering selection lines (Burgess et al., 2007). That is almost exactly three times the 8‐day divergence found in the single generation of selection here. In that population, heritability of flowering time estimated using variance components (*h* ^2 ^= 0.44, Galloway et al., 2009) was also nearly identical to what was found here. In contrast, heritability of germination time (*h* ^2 ^= 0.12) was only about half of the average heritability measured here and resembled values found when selecting both germination and flowering time in the same direction. Although the average values were similar across studies, the realized heritability estimates of each selection treatment reported here (Figure 2) were both higher and lower than those from previous work, suggesting that including trait correlations in the selection process may enhance or retard evolutionary change. In natural environments, selection commonly acts on multiple traits, and therefore, heritability estimates are expected to be more extreme than if selection acted on traits in isolation.

The evolution of earlier phenology was not constrained by correlations between the two traits. Response to selection for early germination did not depend on whether plants were also selected for early flowering or for late flowering. Similarly, response to selection for early flowering did not depend on the direction of selection for timing of germination. This lack of constraint is consistent with a negligible correlation between timing of germination and flowering found in earlier work using a variance component approach (*r*_*A*_ = −0.029, equivalent to *r*_*Total*_ *,* Galloway et al., 2009). Selection in natural populations frequently favors early flowering (Austen, Rowe, Stinchcombe, & Forrest, 2017; Munguía‐Rosas, Ollerton, Parra‐Tabla, & De‐Nova, 2011), a pattern expected to become increasingly common in warm climates (Anderson, Inouye, McKinney, Colautti, & Mitchell‐Olds, 2012), including in *C. americana* (Haggerty & Galloway, 2011). The ability to evolve early flowering, regardless of the pattern of selection on timing of germination, will enhance *C. americana*'s ability to respond to changing climates. However, genetic correlations may constrain selection response to changing climates in other taxa (Etterson & Shaw, 2001).

In contrast, selection in opposite directions on the two traits resulted in a greater evolution of late germination and flowering, and a larger realized heritability. This suggests a negative genetic covariance between the traits, with enhanced response when selection was along the major axis of correlation and less when it was perpendicular to that axis (Conner, 2012). If there is an asymmetric distribution of the covariance between the traits, with greater genetic covariation available for delayed phenology than early traits, it would create a pattern where trait associations were more important for selection to delay phenology than to accelerate it. Selection differentials provide some evidence for this. The extent to which each selected group differed from the population mean, that is, the selection differential, was consistently greater for the late selection treatments than the early selection treatments (late 1.3 and 1.7 times larger than early in the two base populations for germination; 1.6 and 1.1 times larger for flowering). Larger selection differentials suggest greater genetic variation and potentially covariation. In contrast, reduced covariation between the traits for early phenology, indicated by their independent evolution, may reveal a history of selection off the major axis of correlation, such as for early germination and early flowering (e.g., Beldade, Koops, & Brakefield, 2002; Conner et al., 2011; Delph, Steven, Anderson, Herlihy, & Brodie, 2011).

Transgenerational impacts of maternal flowering time on offspring germination season in *C. americana* may affect the genetic correlation between timing of flowering and germination. Earlier flowering mothers produce seeds that are more likely to germinate early and thus grow as annuals, while later flowering mothers produce later germinating seeds that tend to have a biennial life history schedule (Galloway & Burgess, 2009). Environmental correlations drive this transgenerational effect as flowering time determines subsequent reproductive phenology, including timing of seed dispersal, which in turn affects germination season (Galloway, 2002; Galloway & Burgess, 2009). A positive cross‐generation genetic correlation is expected to accelerate the evolution of life history schedule (Kirkpatrick & Lande, 1989). For example, selection for early flowering can lead to an increase in annuals because early flowering is genetically correlated with early germination, but also because early flowering results in earlier seed dispersal and germination. In contrast, a negative cross‐generation genetic correlation between the traits, such as found here when selecting for delayed phenology, will counteract the environmental correlation, slowing the response to selection. This suggests that transgenerational effects constraining evolutionary change (e.g., Räsänen & Kruuk, 2007; Wilson & Réale, 2006) may have shaped the genetic correlation found between germination and flowering time.

As life history schedule in *C. americana* is determined by season of germination, it may evolve through genetic change in the timing of germination, with earlier germination leading to an increase in annuals, or through a change in maternal flowering time, again with earlier flowering resulting in an increase in annual offspring. Therefore, selection favoring an increased frequency of annual *C. americana*, likely to occur as climate change expands the length of the growing season (Vidigal et al., 2016), may act to alter the maternal and/or offspring contribution to life history schedule. Selection is expected to be stronger on earlier developmental traits because they determine the context for subsequent trait expression and therefore may constrain variation (Donohue, 2014; Saltz & Nuzhdin, 2014). For life history schedule in *C. americana*, flowering time determines the context in which germination occurs. For example, late‐dispersed seeds from late‐flowering maternal plants are unlikely to germinate in the fall as annuals due to the onset of cold temperatures, regardless of their genetics for timing of germination. The combined earlier developmental expression and larger heritability of flowering time suggest that life history schedule will evolve largely through changes in this trait. Indeed, the substantial variation among *C. americana* populations in flowering time may reflect selection for its transgenerational effects on life history schedule.

Studying response to selection on phenology in *C. americana* reveals that understanding the evolution of complex phenotypes, and those that are part of a developmental cascade, requires evaluating multiple components that may be targets of selection. Furthermore, it indicates that genetic correlations may vary across the trait distribution resulting in a complicated landscape of trait associations that influence response to selection.

## CONFLICT OF INTEREST

None decalred.

## AUTHORS CONTRIBUTIONS

L.G. and H.P. conceptualized the study and designed the experiment; R.W. and H.P. collected the data; L.G. and R.W. analyzed and interpreted the data; L.G. and R.W. wrote the manuscript, and H.P. contributed to revisions. All authors approved the submitted manuscript.

## DATA ACCESSIBILITY

Data available from the Dryad Digital Repository: https://doi.org/10.5061/dryad.j2m3vm9
