# Supplementary material for: Response to joint selection on germination and flowering phenology depends on the direction of selection
Source: Ecol Evol. 2018 Jul 12;8(15):7688–96. doi: 10.1002/ece3.4334 (PMC6106181; doi:10.1002/ece3.4334)
Supplement: Supplementary file 1 [file ECE3-8-7688-s001.docx]

**Supplemental Table 1**. Location of *Campanula americana* populations crossed to form the base populations for artificial selection: MS55 with MI44 (Base Population I), MS70 with TN34 (Base Population II).

| ID | ID in Prendeville et al. 2013 | State | Latitude | Longitude |
| --- | --- | --- | --- | --- |
| MS55 | MS1 | Mississippi | 31.74407 | -88.52413 |
| MS70 | MS2 | Mississippi | 34.00155 | -88.5776 |
| TN34 | TN | Tennessee | 36.08207 | -86.29612 |
| MI44 | MI2 | Michigan | 42.30103 | -85.35678 |

**Supplemental Table 2**. Realized heritability, *h*^2^, of days to germination and days to flowering in *C. americana* following artificial selection for early germination and early flowering (EE), early germination and late flowering (EL), late germination and early flowering (LE), late germination and late flowering (LL) for two replicate lines each for each base population.

| Base  Population | Selection  Treatment | Germination *h*^2^ (SE) | | | Flowering *h*^2^ (SE) | | |
| --- | --- | --- | --- | --- | --- | --- | --- |
|  |  | Line 1 | Line 2 | Line 1 | | Line 2 | |
| I | EE | 0.19 (0.01) | 0.10 (0.01) | 0.36 (0.02) | | | 0.59 (0.03) |
| II |  | 0.27 (0.02) | 0.00 (0.01) | 0.47 (0.02) | | | 0.46 (0.02) |
| I | EL | 0.27 (0.02) | -0.29 (0.01) | 0.43 (0.01) | | | 0.34 (0.01) |
| II |  | 0.51 (0.03) | 0.27 (0.03) | 0.41 (0.02) | | | 0.47 (0.01) |
| I | LE | 0.71 (0.03) | 0.72 (0.02) | 0.50 (0.04) | | | 0.56 (0.04) |
| II |  | 0.69 (0.03) | 0.49 (0.02) | 0.56 (0.04) | | | 0.39 (0.02) |
| I | LL | 0.12 (0.01) | 0.26 (0.01) | 0.25 (0.01) | | | 0.27 (0.01) |
| II |  | 0.10 (0.00) | 0.29 (0.01) | 0.09 (0.02) | | | 0.31 (0.01) |
